# Euphorbia Drumondii

**Published:** 1887-03

**Authors:** 


Euphorbia Drumondii.—A new alkaloid has been discovered in one of the euphorbiaceæ, *euphorbia Drumondii*, of Australian origin. The alkaloid has been called *drumina* and possesses the anæsthetic properties of cocaine. Cattle and sheep that eat the plant die after being affected with a paralysis of the extremities. A solution of drumina applied to the tongue, nasal membrane, or upon the hands, will constantly produce anæsthesia so complete that the tongue, for example, cannot detect the bitter taste of quinine. Injected subcutaneously in cases of sciatica and neuralgia, it will produce instantaneous relief.

Dr. Ried, the discoverer, believes this alkaloid to be a pure anæsthetic, acting without a preceding stage of excitement, and claims that it can be administered without danger.—*Il Morgagni.*

